# Proteome profiling of clear cell renal cell carcinoma in von Hippel-Lindau patients highlights upregulation of Xaa-Pro aminopeptidase-1, an anti-proliferative and anti-migratory exoprotease

**DOI:** 10.18632/oncotarget.21929

**Published:** 2017-10-19

**Authors:** Vanessa Drendel, Bianca Heckelmann, Chia-Yi Chen, Juliane Weisser, Guadalupe Espadas, Christoph Schell, Eduard Sabido, Martin Werner, Cordula A. Jilg, Oliver Schilling

**Affiliations:** ^1^ Department of Pathology, Medical Center–University of Freiburg, Faculty of Medicine, University of Freiburg, Freiburg, Germany; ^2^ Institute of Molecular Medicine and Cell Research, Faculty of Medicine, University of Freiburg, Freiburg, Germany; ^3^ Present Address: CeMM Research Center for Molecular Medicine of the Austrian Academy of Sciences, Vienna, Austria; ^4^ Proteomics Unit, Centre for Genomic Regulation (CRG), The Barcelona Institute for Science and Technology, Barcelona, Spain; ^5^ Universitat Pompeu Fabra, Barcelona, Spain; ^6^ German Cancer Consortium (DKTK) and German Cancer Research Center (DKFZ), Heidelberg, Germany; ^7^ Department of Urology, Medical Center–University of Freiburg, Faculty of Medicine, University of Freiburg, Freiburg, Germany; ^8^ BIOSS Centre for Biological Signaling Studies, University of Freiburg, Freiburg, Germany

**Keywords:** von Hippel-Lindau disease, clear cell renal cell carcinoma, formalin-fixation, paraffin embedment, proteomics

## Abstract

Patients of the von Hippel-Lindau (VHL) disease frequently develop clear cell renal cell carcinoma (ccRCC). Using archived, formalin-fixed, paraffin-embedded (FFPE) samples, we sought to determine global proteome alterations that distinguish ccRCC tissue from adjacent, non-malignant kidney tissue in VHL-patients. Our quantitative proteomic analysis clearly discriminated tumor and non-malignant tissue. Significantly dysregulated proteins were distinguished using the linear models for microarray data algorithm. In the ccRCC tissue, we noticed a predominant under-representation of proteins involved in the tricarboxylic acid cycle and an increase in proteins involved in glycolysis. This profile possibly represents a proteomic fingerprint of the “Warburg effect”, which is a molecular hallmark of ccRCC. Furthermore, we observed an increase in proteins involved in extracellular matrix organization. We also noticed differential expression of many exoproteases in the ccRCC tissue. Of particular note were opposing alterations of Xaa-Pro Aminopeptidases-1 and -2 (XPNPEP-1 and -2): a strong decrease of XPNPEP-2 in ccRCC was accompanied by abundant presence of the related protease XPNPEP-1. In both cases, we corroborated the proteomic results by immunohistochemical analysis of ccRCC and adjacent, non-malignant kidney tissue of VHL patients. To functionally investigate the role of XPNPEP-1 in ccRCC, we performed small-hairpin RNA mediated XPNPEP-1 expression silencing in 786-O ccRCC cells harboring a mutated *VHL* gene. We found that XPNPEP-1 expression dampens cellular proliferation and migration. These results suggest that XPNPEP-1 is likely an anti-target in ccRCC. Methodologically, our work further validates the robustness of using FFPE material for quantitative proteomics.

## INTRODUCTION

Von Hippel-Lindau (VHL) disease is a rare disease (incidence 1:35 000 - 1:50 000), which results in a variety of tumor syndromes [1, 2]. VHL patients inherit a single mutant *VHL* allele. Tumor development follows somatic inactivating mutations of the remaining wild-type *VHL* allele. Frequently, VHL patients develop clear cell renal cell carcinoma (ccRCC) [2]. Likewise, somatic mutation of the *VHL* gene is also a frequent event in sporadic ccRCC (occurring in non-VHL patients) [3]. The VHL protein (pVHL) is part of a multi protein complex, which functions as a ubiquitin ligase [4] with HIF transcription factors being important substrates Jaakkola, 2001 #82}. Loss of functional pVHL results in the accumulation of HIF transcription factors with subsequent consecutive expression of hypoxia-related genes, affecting metabolic and apoptotic processes as well as vascularization. However, several *in vitro* and *in vivo* studies highlight that *VHL* inactivation alone is insufficient to cause the development of overt malignancies [5–8].

ccRCC has been in the focus of several unbiased expression studies in order to determine key characteristics of the neoplastic malignant tissue and potential targets for therapeutic intervention. This includes both transcriptomic [9–12] and proteomic strategies [13–19]. These studies typically focused on sporadic ccRCC. Beroukhim et al. performed a transcriptomic survey of ccRCC in VHL patients [9]. System-wide analysis of proteins (“proteomics”) is gaining interest for the investigation of malignancies due to the limited correlation between mRNA and protein levels [20, 21]. Formalin-fixed, paraffin-embedded (FFPE) samples are increasingly recognized as a robust and valuable resource for proteomic profiling [22–24]. FFPE samples constitute the predominant form of tissue storage in most clinico-pathological archives, especially for collections that have been assembled over decades. This aspect renders the investigation of FFPE specimens particularly useful for research on rare diseases and with a long time follow-up. In the present study, we employed “FFPE proteomics” for the proteomic profiling of ccRCC tissue from VHL patients compared to patient-matched, non-malignant kidney tissue. Amongst other findings, we observe a prototypical proteomic fingerprint of aerobic glycolysis (“Warburg effect”) in ccRCC tissue together with differential regulation of Xaa-Pro Aminopeptidases-1 and -2 (tumor-specific increase of XPNPEP1 with concomitant decrease of XPNPEP2).

## RESULTS AND DISCUSSION

### Experimental set-up

We aimed for a proteome characterization of ccRCC in VHL patients. We employed FFPE samples since they represent the prevalent form of storage of clinical specimens, especially for rare diseases. We have recently shown that FFPE samples are amenable to quantitative proteomic analysis using isotope-coded dimethylation [22] and we employed this technique for the present study. Our cohort comprised eight cases; in each case we compared the proteome of ccRCC tissue to the adjacent non-malignant tissue.

In order to enable cohort-wide comparison, we also included a pooled sample (comprised of ccRCC and non-malignant tissue) as a spike-in reference standard against which every other sample can be compared. This set-up is reminiscent of the Super-SILAC technique, in which metabolically labelled proteomes serve as spike-in reference standard [25].

**Figure 1:**
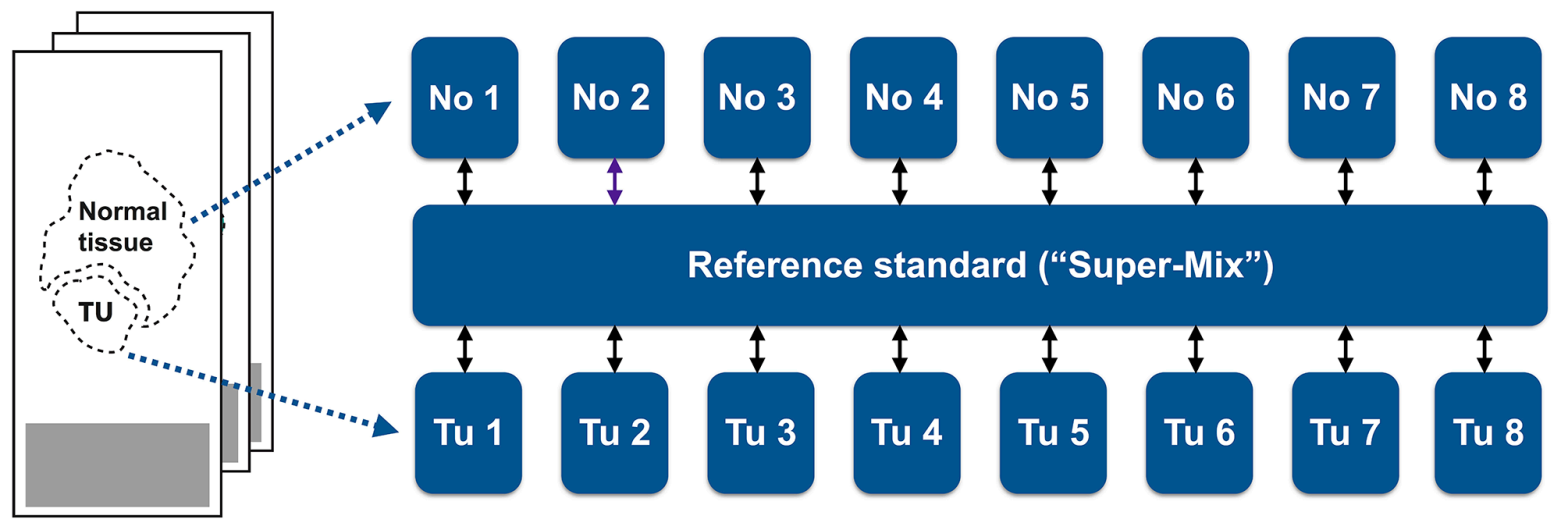
Patient matched samples of ccRCC tissue and adjacent non-malignant tissue were collected from FFPE specimens and, post-trypsination, differentially labeled by isotopic, formaldehyde-based dimethylation A differentially labeled, pooled reference standard was also included.

With tumor tissue, non-malignant tissue, and the pooled standard, a triplex labeling scheme was employed (Figure 1).

### Proteome profile of ccRCC in VHL patients

**Figure 2:**
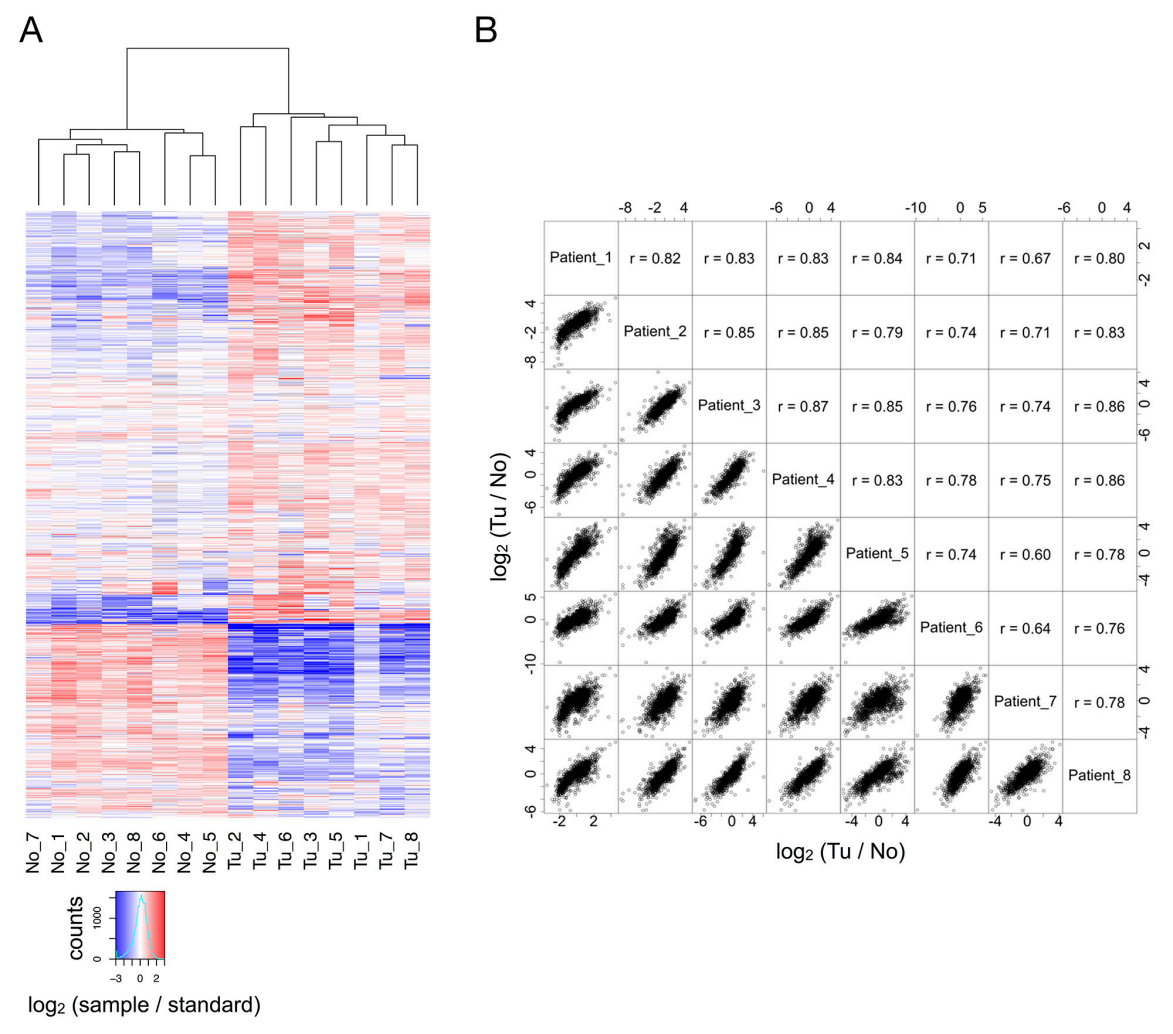
**(A)** In comparison to the reference standard, tumor and non-malignant tissue are clearly separated by hierarchical clustering. **(B)** For all eight cases, there is good correlation between the quantitative alterations observed in direct comparison of tumor and non-malignant tissue.

LC-MS/MS analysis enabled the identification (at a false discovery rate < 1 %) and quantitation of 1716 proteins that were identified in at least six of the eight cases; thus representing a robust and confident survey of the ccRCC proteome and its non-malignant counterpart. As outlined above, inclusion of a pooled standard enables cohort-wide comparison. In unsupervised hierarchical clustering, the individual profiles of the tumor and “normal” tissue samples, when compared to the pooled standard, were clearly separated, resulting in unambiguous clusters that represent either ccRCC tissue or adjacent, non-malignant tissue (Figure 2A). This result validates our experimental approach.

Our triplex labeling strategy further enabled the direct quantitative comparison of the patient-matched samples of renal cancer tissue and adjacent non-malignant tissue. The resulting profiles of quantitative proteome differences evidence a high degree of similarity (Figure 2B).

These findings highlight limited heterogeneity in the cancer and non-malignant tissues of the different cases, despite differences in the clinical progression of the diseases, e.g. with regard to recurrence. A potential caveat, albeit faced by most genomic, transcriptomic, and proteomic investigations, is that decisive proteome differences of individual cells may be overshadowed by bulk sample material.

### Quantitative proteome differences between renal cancer tissue and non-malignant kidney tissue

**Figure 3:**
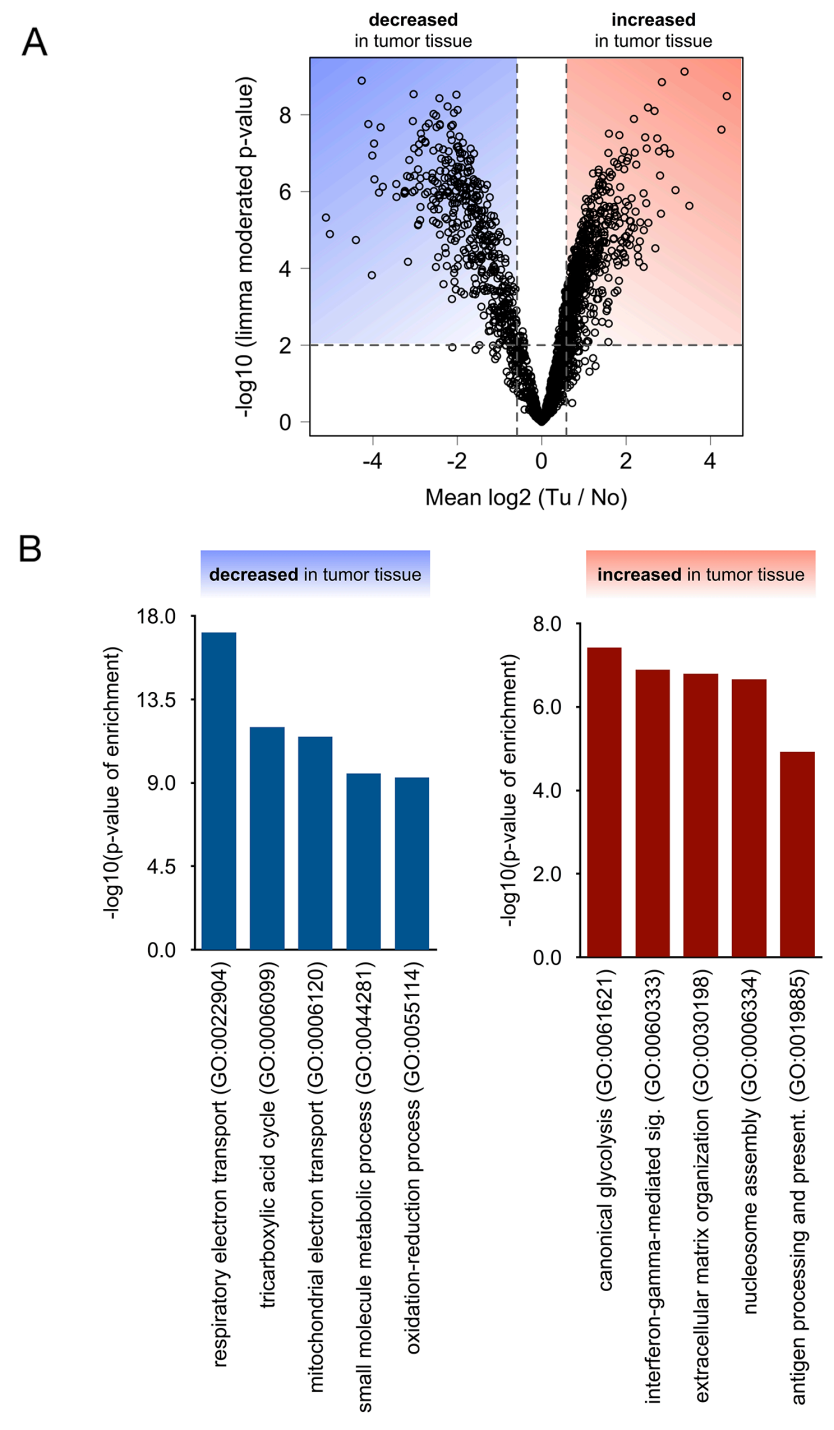
**(A)** Statistical analysis using linear models as implemented in Limma distinguishes significantly (p < 0.01) affected proteins. **(B)** Gene ontology (confined to “biological processes”) analysis of proteins that were found to be significantly decreased or increased in tumor tissue.

To identify proteins that are significantly enriched or depleted in renal cancer tissue as compared to adjacent, non-malignant tissue, we employed a linear model as implemented in the limma statistical package [26], which is particularly powerful with regard to multiple testing correction and prevention of false-positive discoveries in the analysis of omics-style data. Omics-style data frequently suffers from a comparably small number of biological replicates (here: 8 cases) but a large number of analytes (here: > 1700 quantified proteins). We chose the following criteria to identify proteins that exhibit a significant change in abundance: (a) limma moderated p-value < 0.01 and (b) average increase or decrease in abundance by more than 50 % (log_2_ (Tu / No) > 0.58 for increase; log_2_ (Tu / No) < -0.58 for decrease). These criteria resulted in 341 down-regulated proteins in the renal cancers and 267 up-regulated proteins in the renal cancers. The corresponding volcano plot is visualized in Figure 3A; limma moderated p-values and average log2 ratios are listed in Supplementary Table 1.

Decreased levels of the protease neprilysin (cluster of differentiation (CD) 10, synonyms are membrane metallo-endopeptidase (MME), neutral endopeptidase (NEP)) have been observed in numerous studies on ccRCC and our results faithfully replicate this characteristic feature. Indeed., in comparison to the adjacent non-malignant tissue, there is > 4-fold decrease of neprilysin in the renal cancer tissue (average log_2_ Tu / No 1 = -2.76; p_limma_ < 0.01). Numerous proteome or transcriptome corroborate a notable decrease of neprilysin in renal cancer [10, 17, 27, 28], which is consistent with our experimental results.

### Biological processes and pathways affected in renal cancer

In order to functionally classify the differentially regulated proteins in ccRCC tissue and to identify clusters of co-regulated, functionally related proteins, we performed a gene ontology (GO) enrichment analysis, with a focus on the “biological process” annotation [29, 30]. We chose the TopGO algorithm to minimize GO term redundancy [31–33]. Only clusters with a p-value < 0.01 and including at least four significantly affected proteins were considered. Using the “biological process” process annotation, this approach identified several clusters, that are either enriched or depleted in renal cancer tissue as compared to adjacent, non-malignant tissue (Figure 3B). Enriched biological processes comprised aerobic glycolysis, immune processes such as interferon γ signaling, and reorganization of the extracellular matrix, whereas depleted biological processes mostly comprised mitochondrial respiration and its associated processes.

In our proteome characterization of ccRCC in VHL patients we observed a Warburg-type metabolic remodelling, which is widely considered a hallmark feature of ccRCC [3, 34] as well as being reminiscent of hypoxia-type expression regulation. Notably, we also found elevated levels of nearly all enzymes of canonical glycolysis to convert glucose-6-phosphate into pyruvate and lactate; namely glucose-6-phosphate isomerase, 6-phosphofructokinase, fructose-bisphosphate aldolase, triosephosphate isomerase, glyceraldehyde-3-phosphate dehydrogenase, phosphoglycerate kinase, phosphoglycerate mutase, pyruvate kinase, and lactate dehydrogenase (Supplementary Figure 2).

In addition to the “Warburg” profile, further examples of hypoxia-type expression regulation include upregulation of pro-angiogenic proteins such as galectin-3 [35] and MUC18 [36]. Major vault protein is known to be upregulated in hypoxia [37] and likely interacts directly with hypoxia-inducible factor (HIF)-α [38].

Another, upregulated protein cluster comprised proteins that are part of the extracellular matrix or that participate in cell-matrix interactions. Although this cluster was less prominent than the Warburg-effect in ccRCC, a large number of studies corroborate a similar proteome enrichment. Prominent examples are the matricellular protein periostin [13, 19, 39] and fibronectin [16, 17, 40, 41]. Increased levels of type-VI collagen in ccRCC have also been corroborated elsewhere [11] as is the case for integrin β-2 [13]. Similarly, a large body of literature supports elevated levels of annexin A2 in ccRCC [18, 22, 42–44]. We conclude that extracellular matrix components and proteins involved in cell-matrix interaction constitute important proteomic themes of ccRCC.

### Differential expression of Xaa-Pro aminopeptidases in renal cancer

We noticed differential expression of a surprisingly large number of exoproteases in the ccRCC tissue. We use the term “exoproteases” in a wider sense, covering also di- and tri-peptidases; hence collectively referring to proteolytic enzymes that remove one, two or three residues from the amino- or carboxy-termini of proteins.

Exoproteases exhibiting an increase in abundance (as compared to adjacent non-malignant tissue) were aspartyl aminopeptidase, tripeptidyl-peptidase (TPP-1), cytosolic non-specific dipeptidase (CNDP2), endoplasmic reticulum aminopeptidase 1, and Xaa-Pro aminopeptidase 1 (XPNPEP1).

In contrast, exoproteases showing a decrease in abundance (as compared to adjacent non-malignant tissue) were Xaa-Pro aminopeptidase 2 (XPNPEP2), acylamino-acid-releasing enzyme, carboxypeptidase Q, dipeptidyl peptidase 1 (Cathepsin C), carboxypeptidase C (Cathepsin A), aminopeptidase N, renal dipeptidases, and Xaa-Pro dipeptidases.

For some of the aforementioned proteins, further omics-style studies on ccRCC corroborate our findings. Examples include CNDP2 [16, 17, 42], TPP-1 [16], aminopeptidase N [16, 17], and cathepsin C [45]. However, the collective and substantial alteration of exopeptidase abundance in ccRCC has so far been only rarely discussed in the corresponding literature, although tumor-specific alterations in exoproteolytic activity patterns have been noted [46]. Importantly, while (endo-)proteolytic enzymes are typically produced as inactive zymogens that require further activation, this is less often the case for exopeptidases. Many exopeptidases are synthesized as catalytically active enzymes without the requirement of further activation. Secondly, regulation by endogenous inhibitors is less prominent for exo- than for many endoproteases. Without these two levels of auxiliary activity regulation, altered abundance of such exoproteases likely represents altered exoproteolytic activity in a direct manner.

**Figure 4:**
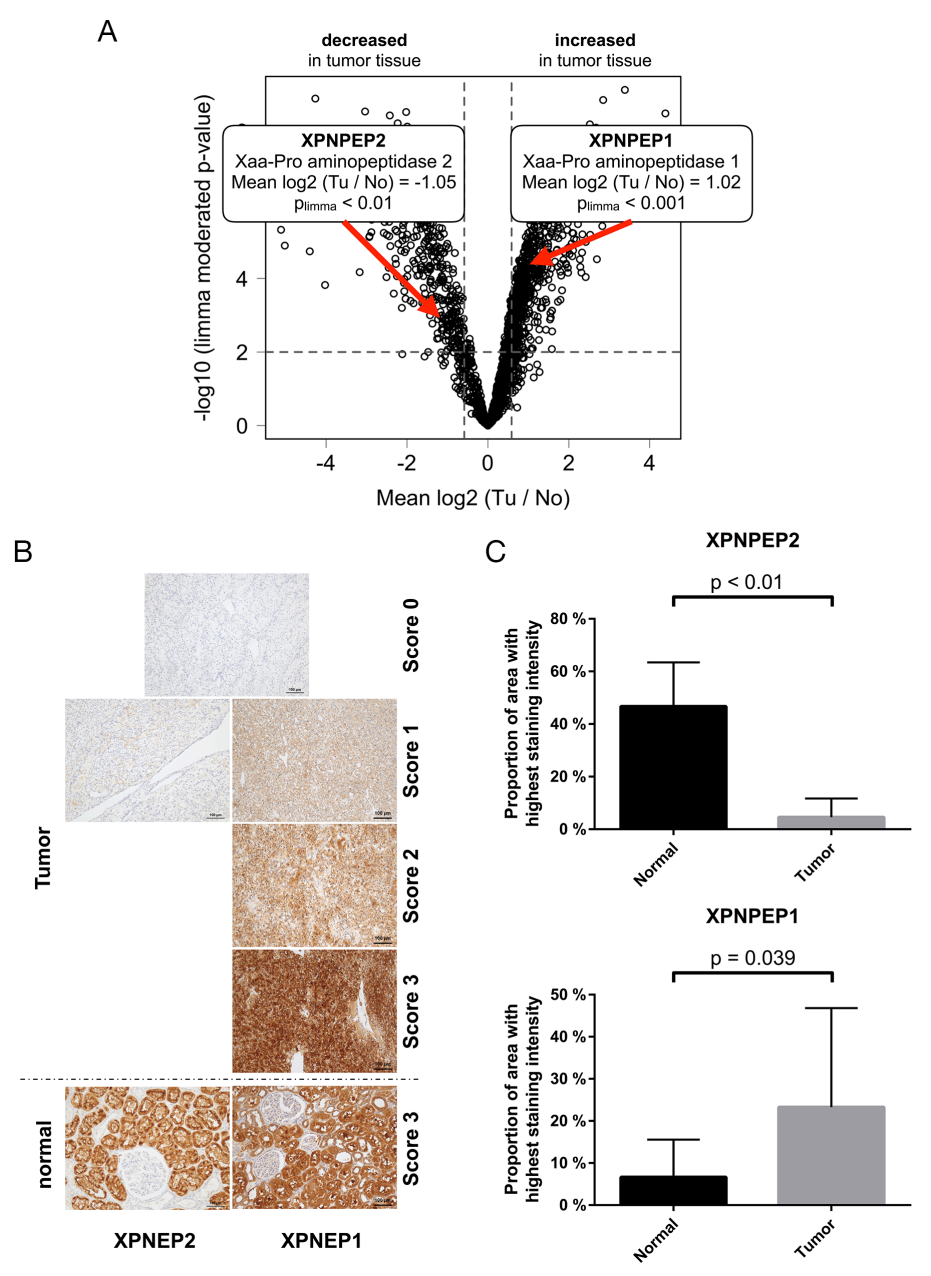
**(A)** The exoproteases XPNPEP1 and -2 are regulated, on the protein level, in opposing directions in ccRCC tissue **(B)** exemplary immunohistochemical staining intensities of XPNPEP1 and -2 **(C)** Immunohistochemical analysis of XPNPEP1 and -2 corroborates the proteomic analysis. p-value refers to two-sided student t-test, p < 0.05).

Out of the array of differentially regulated exoproteases, we chose to further study Xaa-Pro aminopeptidases -1 and -2 (XPNPEP1 and -2, respectively, Figure 4A) since their tumor-contextual expression has been rarely investigated. XPNPEP1 is a cytosolic enzyme while XPNPEP2 is glycophosphatidylinositol (GPI) anchored on the cytosolic side of the plasma membrane. Both enzymes are metallopeptidases that use manganese as a cofactor for activity. XPNPEP1 and -2 are thought to have comparable structural properties and similar substrate specificities [47]. It is however unknown, whether they can functionally compensate for each other. Cleaving after proline (“P1 proline” in Schechter-Berger nomenclature [48]) XPNPEP1 and -2 remove di- or tri-peptides from protein or peptide N-termini. XPNPEP1 and -2 have been mostly studied with regard to their ability of processing bradykinin [49, 50] *in vitro* (see also below). We found very few reports of XPNPEP1 or -2 in cancer biology. One study associates XPNPEP1 with unfavorable outcome in acute myeloid leukemia [51]. We are not aware of any studies on the role of XPNPEP proteases in the context of VHL disease or renal cancer.

We further employed immunohistochemistry (IHC) to investigate the expression of XNPEP1 and -2 in ccRCC of VHL patients, using the same cohort employed for the proteomic study. In normal, non-malignant kidney tissue, we observed abundant expression of XPNPEP1 and XPNPEP2 in proximal tubuli (Figure 4B; also supported by published reports on XPNPEP1 activity [52]). In ccRCC tissue, there is only scant detection of XPNPEP2 but strong presence of XPNPEP1 (Figure 4B). We notice significantly decreased levels of XPNPEP2 (p < 0.01, two-sided student t-test, Figure 4C) as well as significantly elevated levels of XPNPEP1 (p < 0.05, two-sided student t-test, Figure 4C) in the ccRCC tissue. In ccRCC, XPNPEP1 is predominantly expressed by the actual tumor cells. Of note however, we employed macrodissection in order to focus on tumor tissue with few other cell types (e.g. fibrotic areas). We conclude that the IHC analysis corroborates the initial quantitative proteomic findings. To our knowledge, this is the first dedicated report of differential regulation of XPNPEP proteases in ccRCC.

The present study focused on ccRCC in VHL disease patients. Many of these patients suffer form hypertension [53, 54]. Likewise, some studies suggest that renal cancers secrete vasoactive factors, which may lead to hypertension [55]. On the proteomic level, the finding of hypertensive factors being secreted by renal cancers is somewhat contrasted by the hallmark decrease of neprilysin, which degrades hypotensive peptides, including kinins (i.e. bradykinin) and atrial natriuretic factor [56]. XPNPEP1, which we found to be upregulated in ccRCC, has been reported to contribute to the degradation of bradykinin. However, this annotation is essentially based on *in vitro* cleavage assays [57] that fail to account for the cytosolic localization of XPNPEP1, while bradykinin is an extracellular peptide. We conclude that the prominent role of exo- and endoproteases in regulating vasoactive peptides together with their differential regulation in ccRCC is suggestive of a link to impaired blood pressure control; however a clear picture fails yet to emerge.

### XPNPEP-1 restricts renal cancer cell proliferation and migration

**Figure 5:**
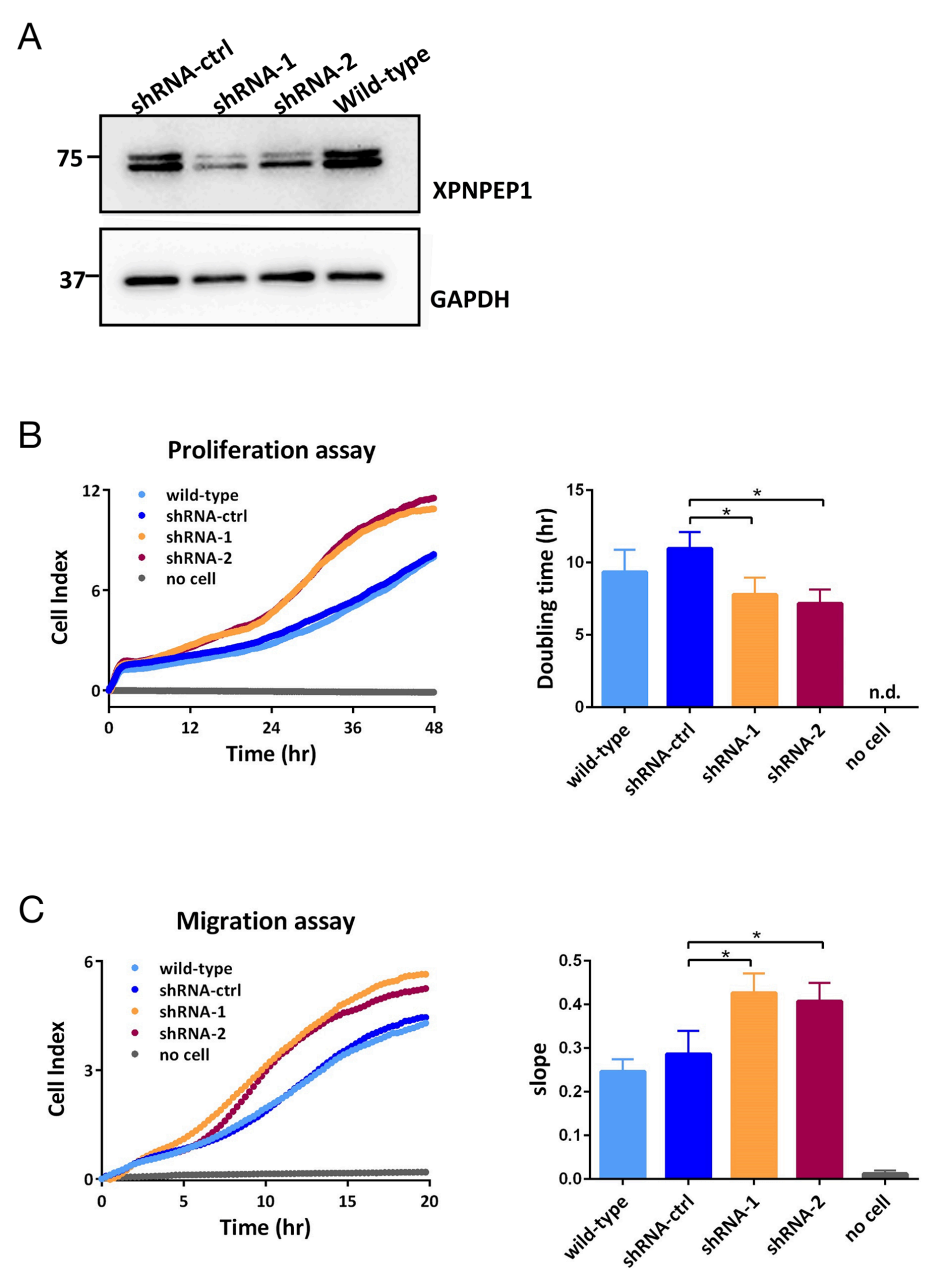
**(A)** Downregulation of XPNPEP1 in 786-O cells by stable transduction with small-hairpin (sh)RNAs as detected by immunoblotting. **(B, C)** Reduced expression of XPNPEP1 leads to enhanced cell proliferation (B) and migration (C) as determined by XCELLigence real time monitoring with data points taken every 15 min. Unpaired Student t-test with two-tailed p-value was employed for statistical analysis; ^*^ denotes p < 0.05. The individual data points of the XCELLigence data are shown. n.d., not detected.

The strong presence of XPNPEP1 in ccRCC motivated us to investigate its impact on cancer cell functionality. To this end, we chose the human renal cell adenocarcinoma cell line 786-O, which contains a mutated *VHL* gene. 786-O cells are frequently used as cellular model systems to study functional aspects of renal cancer *in vitro* [58–60]. In line with 786-O cells being a cancer cell line, we found noticeable expression levels of XPNPEP1 (Figure 5A). Four isoforms of XPNPEP1 are being reported, with sequence lengths ranging from 599 to 666 amino acids. By immunoblotting, we detected two protein species in the range of 70 – 75 kDa, which corresponds well to the predicted molecular weights of the different XPNPEP1 isoforms. Although the occurrence of two bands in the immunoblotting analysis may indicate the presence of different XPNPEP1 isoforms, it may also be attributed to differential post-translational processing such as proteolytic truncation or acetylation. We emphasize that our data does not unambiguously demonstrate the occurrence of different XPNPEP1 isoforms in 786-O cells. We suppressed XPNPEP1 expression by RNA interference (RNAi), using stable transduction with two different small hairpin RNAs (shRNAs) and, as a control, a non-targeting shRNA. As highlighted in Figure 5A, we achieved marked reduction of XPNPEP1 expression. Functional characterization indicated that reduced XPNPEP1 expression consistently resulted in mildly increased cell proliferation (i.e. shorter doubling time) and increased chemotactic migration (Figure 5B). Since proliferation and migration are integral features of the malignant potential of cancer cells, our *in vitro* data suggests a rather anti-tumorigenic function of XPNPEP1, despite its strong upregulation in renal cancer. However, we have only probed a small number of functional aspects (i.e. proliferation and migration) and cannot exclude the possibility of a pro-tumorigenic impact on further aspects of tumor biology, such as metabolism or tumor-stroma interaction, to name just a few examples. Nevertheless, the seemingly paradoxical situation of abundant, tumor-specific expression together with a rather anti-tumorigenic functionality has also been observed for other proteases, such as several matrix metalloproteases, which are now considered as therapeutic anti-targets [61].

## MATERIALS AND METHODS

### Ethics statement

The study was approved by the Ethics Committee of the University Medical Center Freiburg (311/12). Before study inclusion, all patient data were anonymized.

### Tissue collection and sample preparation

FFPE tissue specimens of clear cell renal cell carcinoma (ccRCC) and adjacent non-malignant kidney tissue were chosen. As outlined above, all tissues stem from 8 VHL patients. Necrotic and fibrotic areas as well as areas of hemorrhage and inflammation, which are common findings in kidney cancers, were marked under the light microscope on an HE stained slide (Supplementary Figure 1) and removed by macrodissection from at least three following unstained sections, each of 5 μm thickness. Sample preparation, including deparaffinization before macrodissection and subsequent heat incubation, and trypsination was essentially performed as described previously [22]. For quantitative comparison, triplex isotopic dimethylation of primary amines was employed [62], distinguishing tumor tissue (Tu), adjacent non-malignant tissue (No), and a pooled mix that serves as a standard similar to the Super-SILAC approach [25]. Samples were further fractionated by miniaturized strong cation exchange chromatography (Empore cation-SR, 3M, St. Paul, MN, USA) with a procedure adapted from Rappsilber et al [63]. The peptides were eluted with six different concentrations of ammonium acetate (40, 80, 120, 160, 200 and 500mM). Each fraction was cleaned up on a homemade Empore C18 column (3M, St. Paul, MN, USA) as described by Rappsilber

### LC-MS/MS and data analysis

LC-MS/MS was performed using an Orbitrap Velos Pro mass spectrometer (Thermo Fisher Scientific, San Jose, CA, USA) coupled to an EasyLC 1000 (Thermo Fisher Scientific (Proxeon), Odense, Denmark). Peptides were separated by reversed-phase chromatography using a 12-cm column with an inner diameter of 75 μm, packed with 5 μm C18 particles (Nikkyo Technos Co., Ltd. Japan). Chromatographic gradients started at 97% buffer A and 3% buffer B with a flow rate of 300 nl/min, and gradually increased to 93% buffer A and 7% buffer B in 1 min, to 65% buffer A and 35% buffer B in 120 min. After each analysis, the column was washed for 10 min with 10% buffer A and 90% buffer B. Buffer A: 0.1% formic acid in water. Buffer B: 0.1% formic acid in acetonitrile.

The mass spectrometer was operated in positive ionization mode with nanospray voltage set at 2.2 kV and source temperature at 250 °C. Ultramark 1621 for the FT mass analyzer was used for external calibration prior the analyses. Moreover, an internal calibration was also performed using the background polysiloxane ion signal at m/z 445.1200. The instrument was operated in data-dependent analysis (DDA) mode and full MS scans with 1 micro scan at a resolution of 60,000 were used over a mass range of m/z 350-2000 with detection in the Orbitrap. Automatic gain control (AGC) was set to 1E6, dynamic exclusion was set to 60 s and the charge state filtering disqualifying singly charged peptides was activated. In each DDA cycle, a survey scan (MS1) was recorded and then the top ten most intense ions above a threshold ion count of 5000 were selected for fragmentation at normalized collision energy of 35% (MS2). Fragment ion spectra produced via collision-induced dissociation (CID) were analyzed in the linear ion trap, AGC was set to 5e4, isolation window of 2.0 m/z, activation time of 0.1 ms and maximum injection time of 100 ms was used. All data were acquired with Xcalibur software v2.2.

MS data were analyzed by MaxQuant version 1.5.28 [64] with the Uniprot human database downloaded on November 2015, counting 20193 reviewed entries [65]. The analysis included an initial search with a precursor mass tolerance of 20 ppm for mass recalibration and a main search with precursor mass and fragment mass tolerances of 6 ppm and 20 ppm, respectively. The search included a fixed modification of carbamidomethyl cysteine and no variable modifications. Tryptic cleavage specificity with no missed cleavages was used with a minimal peptide length of seven amino acids. The false discovery rate (FDR) was set to < 1% for peptide and protein identifications in individual analyses. For quantitative comparison between samples we used the amino-dimethylation labeling scheme based on multiplicity three.

Proteins were only further considered if they were identified and quantified in at least six of the eight patient cases. Due to this strict requirement of six biological replicate, we also included proteins that were identified and quantified by single peptides in individual replicates. Files obtained by MaxQuant were further processed using RStudio v.0.99.446 (R Foundation for Statistical Computing, Vienna, Austria) as previously described [66]. Reverse and potential contaminants entries were removed. Ratios were log_2_ transformed, normalized by centering, and a linear model was fitted using the limma package [67].

### Immunohistochemical analysis

For immunohistochemical analysis of XPNPEP1 and XPNPEP2 from selected suitable FFPE tissue samples, slices of 2 μm thickness were prepared using the Leica RM2255 microtome. For XPNPEP2, antigen retrieval was performed for 5 minutes using citrate buffer pH 6,0 using a pressure cooker. For staining of XPNPEP1, no antigen retrieval was necessary. Next, incubation with primary antibodies was conducted (XPNPEP1: monoclonal murine antibody ab123929 (ABCAM); XPNPEP2: polyclonal goat antibody AF2490 (RnD systems)). Incubation times and dilution were 60 min diluted 1:400 in Zytomed dilution buffer (ZUC025-500) for the XPNPEP1 antibody and 60 min diluted 1:450 in Zytomed dilution buffer (ZUC025-500) for the XPNPEP2 antibody. Visualization was performed using DAKO Envision Flex+, Mouse, high pH (Link) Detecting System (K800221-2). Sections were counterstained with hematoxylin for one minute, dehydrated in an ascending alcohol concentration and covered with xylol and coverslipping film (Tissue-Tek^R^ 4770). For evaluation, two experienced pathologists reviewed XPNPEP1 and -2 expression in ccRCC tumor cells and adjacent, non-malignant tissue. Using 50-fold and then 100-fold magnification, XPNPEP1 and -2 expression was analyzed performing a semi-quantitative expression analyses by evaluating XPNPEP1 and -2 intensity (range 0 to 3). In detail, if no staining was detectable the XPNPEP1 or -2 expression was scored negative (score 0). If a mild immunoreactivity was detectable score 1 (low intensity) was used. If XPNPEP1 or -2 was expressed similar to the staining intensity of proximal tubuli of adjacent non-malignant renal tissue than core 3 (high intensity) was assigned. Score 2 (moderate intensity) was used if XPNPEP1 or -2 intensity was weaker than score 3 but more intense than score 1. For statistical analysis of immunohistochemistry, which is based on a two-sided Student t-test, for every patient only the score with the highest percentage of area was included.

### Cell culture, XPNPEP1 knock-down, immunoblotting, and functional analysis

The human renal cell adenocarcinoma cell line 786-O, which contains a mutated *VHL* gene, was purchased from Cell Line Services (Heidelberg, Germany) and cultured in Dulbecco’s modified Eagle’s medium (PAN) supplemented with 10 % fetal calf serum (PAN), 1 % non-essential amino acids, 1 % MEM vitamins and 1 % penicillin/streptomycin (all Gibco/Invitrogen) at 37 °C in humidified air, containing 5 % CO_2_. To reduce the expression of XPNPEP1, three different shRNA constructs were used: shRNA-ctrl (non-targeting shRNA, SHC002), shRNA-1 (TRCN 0000073927), shRNA-2 (TRCN 0000073923) (all Sigma-Aldrich). Viral transduction and selection of stable transfectants was performed as described [68]. Immunoblotting was performed as described [69] using the primary XPNPEP1 antibody ab123929 (ABCAM). Proliferation and migration assays were performed using the xCELLigence System (Roche, Mannheim, Germany) as described [70]. For the migration assay, 20K cells were cultured in CIM plates with serum-free medium in the upper chamber and normal growth medium in the lower chamber. For the proliferation assay, 5K cells were seeded in E-16 plates to monitor the cell doubling rate.

### Data availability

The mass spectrometry proteomics data have been deposited to the ProteomeXchange Consortium via the PRIDE [71] partner repository with the dataset identifier PXD005710.

## CONCLUSION

We present one of the first proteomic profiling studies of ccRCC in VHL patients, in which we found prototypical hallmarks of ccRCC, including a proteomic fingerprint of the Warburg effect and an impact of matricellular proteins and components of the extracellular matrix. Methodologically, our work further validates the robustness of using FFPE material for quantitative proteomics and highlights the important alterations in protein abundance exhibited by several exoproteases, from which we verified by immunohistochemistry for XPNPEP1 and -2. Despite the high abundance of XPNPEP1 in ccRCC tumor tissue, functional assays for XPNPEP1 suggest an anti-proliferative and anti-migratory role for this protein, thus characterizing XPNPEP1 as a putative “anti-target” for ccRCC.

## SUPPLEMENTARY MATERIALS FIGURES AND TABLE





## Abbreviations

ccRCC: clear cell renal cell carcinoma
CID: collision-induced dissociation
DDA: data-dependent analysis
FFPE: formalin-fixed, paraffin-embedded
IHC: immunohistochemistry
MME: membrane metallo-endopeptidase
No: adjacent non-malignant tissue
Tu: tumor tissue
VHL: Von Hippel-Lindau (VHL)

## References

[1] Gossage L, Eisen T, Maher ER (2015). VHL, the story of a tumour suppressor gene. Nat Rev Cancer.

[2] Karsdorp N, Elderson A, Wittebol-Post D, Hene RJ, Vos J, Feldberg MA, van Gils AP, Jansen-Schillhorn van Veen JM, Vroom TM, Hoppener JW, Lips CJM (1994). Von Hippel-Lindau disease: new strategies in early detection and treatment. Am J Med.

[3] Frew IJ, Moch H (2015). A clearer view of the molecular complexity of clear cell renal cell carcinoma. Annu Rev Pathol.

[4] Maxwell PH, Wiesener MS, Chang GW, Clifford SC, Vaux EC, Cockman ME, Wykoff CC, Pugh CW, Maher ER, Ratcliffe PJ (1999). The tumour suppressor protein VHL targets hypoxia-inducible factors for oxygen-dependent proteolysis. Nature.

[5] Mack FA, Rathmell WK, Arsham AM, Gnarra J, Keith B, Simon MC (2003). Loss of pVHL is sufficient to cause HIF dysregulation in primary cells but does not promote tumor growth. Cancer Cell.

[6] Mandriota SJ, Turner KJ, Davies DR, Murray PG, Morgan NV, Sowter HM, Wykoff CC, Maher ER, Harris AL, Ratcliffe PJ, Maxwell PH (2002). HIF activation identifies early lesions in VHL kidneys: evidence for site-specific tumor suppressor function in the nephron. Cancer Cell.

[7] Ma W, Tessarollo L, Hong SB, Baba M, Southon E, Back TC, Spence S, Lobe CG, Sharma N, Maher GW, Pack S, Vortmeyer AO, Guo C (2003). Hepatic vascular tumors, angiectasis in multiple organs, and impaired spermatogenesis in mice with conditional inactivation of the VHL gene. Cancer Res.

[8] Hong SB, Furihata M, Baba M, Zbar B, Schmidt LS (2006). Vascular defects and liver damage by the acute inactivation of the VHL gene during mouse embryogenesis. Lab Invest.

[9] Beroukhim R, Brunet JP, Di Napoli A, Mertz KD, Seeley A, Pires MM, Linhart D, Worrell RA, Moch H, Rubin MA, Sellers WR, Meyerson M, Linehan WM (2009). Patterns of gene expression and copy-number alterations in von-hippel lindau disease-associated and sporadic clear cell carcinoma of the kidney. Cancer Res.

[10] Sato Y, Yoshizato T, Shiraishi Y, Maekawa S, Okuno Y, Kamura T, Shimamura T, Sato-Otsubo A, Nagae G, Suzuki H, Nagata Y, Yoshida K, Kon A (2013). Integrated molecular analysis of clear-cell renal cell carcinoma. Nat Genet.

[11] Wan F, Wang H, Shen Y, Zhang H, Shi G, Zhu Y, Dai B, Ye D (2015). Upregulation of COL6A1 is predictive of poor prognosis in clear cell renal cell carcinoma patients. Oncotarget.

[12] Yusenko MV, Kuiper RP, Boethe T, Ljungberg B, van Kessel AG, Kovacs G (2009). High-resolution DNA copy number and gene expression analyses distinguish chromophobe renal cell carcinomas and renal oncocytomas. BMC Cancer.

[13] Atrih A, Mudaliar MA, Zakikhani P, Lamont DJ, Huang JT, Bray SE, Barton G, Fleming S, Nabi G (2014). Quantitative proteomics in resected renal cancer tissue for biomarker discovery and profiling. Br J Cancer.

[14] Higgins JP, Shinghal R, Gill H, Reese JH, Terris M, Cohen RJ, Fero M, Pollack JR, van de Rijn M, Brooks JD (2003). Gene expression patterns in renal cell carcinoma assessed by complementary DNA microarray. Am J Pathol.

[15] Jones J, Otu H, Spentzos D, Kolia S, Inan M, Beecken WD, Fellbaum C, Gu X, Joseph M, Pantuck AJ, Jonas D, Libermann TA (2005). Gene signatures of progression and metastasis in renal cell cancer. Clin Cancer Res.

[16] Okamura N, Masuda T, Gotoh A, Shirakawa T, Terao S, Kaneko N, Suganuma K, Watanabe M, Matsubara T, Seto R, Matsumoto J, Kawakami M, Yamamori M (2008). Quantitative proteomic analysis to discover potential diagnostic markers and therapeutic targets in human renal cell carcinoma. Proteomics.

[17] Perroud B, Ishimaru T, Borowsky AD, Weiss RH (2009). Grade-dependent proteomics characterization of kidney cancer. Mol Cell Proteomics.

[18] Raimondo F, Salemi C, Chinello C, Fumagalli D, Morosi L, Rocco F, Ferrero S, Perego R, Bianchi C, Sarto C, Pitto M, Brambilla P, Magni F (2012). Proteomic analysis in clear cell renal cell carcinoma: identification of differentially expressed protein by 2-D DIGE. Mol Biosyst.

[19] Zhao Z, Wu F, Ding S, Sun L, Liu Z, Ding K, Lu J (2015). Label-free quantitative proteomic analysis reveals potential biomarkers and pathways in renal cell carcinoma. Tumour Biol.

[20] Shankavaram UT, Reinhold WC, Nishizuka S, Major S, Morita D, Chary KK, Reimers MA, Scherf U, Kahn A, Dolginow D, Cossman J, Kaldjian EP, Scudiero DA (2007). Transcript and protein expression profiles of the NCI-60 cancer cell panel: an integromic microarray study. Mol Cancer Ther.

[21] Wilhelm M, Schlegl J, Hahne H, Gholami AM, Lieberenz M, Savitski MM, Ziegler E, Butzmann L, Gessulat S, Marx H, Mathieson T, Lemeer S, Schnatbaum K (2014). Mass-spectrometry-based draft of the human proteome. Nature.

[22] Weisser J, Lai ZW, Bronsert P, Kuehs M, Drendel V, Timme S, Kuesters S, Jilg CA, Wellner UF, Lassmann S, Werner M, Biniossek ML, Schilling O (2015). Quantitative proteomic analysis of formalin-fixed, paraffin-embedded clear cell renal cell carcinoma tissue using stable isotopic dimethylation of primary amines. BMC Genomics.

[23] Bronsert P, Weisser J, Biniossek ML, Kuehs M, Mayer B, Drendel V, Timme S, Shahinian H, Kusters S, Wellner UF, Lassmann S, Werner M, Schilling O (2014). Impact of routinely employed procedures for tissue processing on the proteomic analysis of formalin-fixed paraffin-embedded tissue. Proteomics Clin Appl.

[24] Gustafsson OJ, Arentz G, Hoffmann P (2015). Proteomic developments in the analysis of formalin-fixed tissue. Biochim Biophys Acta.

[25] Geiger T, Cox J, Ostasiewicz P, Wisniewski JR, Mann M (2010). Super-SILAC mix for quantitative proteomics of human tumor tissue. Nat Methods.

[26] Smyth GK (2004). Linear models and empirical bayes methods for assessing differential expression in microarray experiments. Stat Appl Genet Mol Biol.

[27] Guo T, Kouvonen P, Koh CC, Gillet LC, Wolski WE, Rost HL, Rosenberger G, Collins BC, Blum LC, Gillessen S, Joerger M, Jochum W, Aebersold R (2015). Rapid mass spectrometric conversion of tissue biopsy samples into permanent quantitative digital proteome maps. Nat Med.

[28] Varona A, Blanco L, Perez I, Gil J, Irazusta J, Lopez JI, Candenas ML, Pinto FM, Larrinaga G (2010). Expression and activity profiles of DPP IV/CD26 and NEP/CD10 glycoproteins in the human renal cancer are tumor-type dependent. BMC Cancer.

[29] Gene Ontology Consortium (2015). Gene Ontology Consortium: going forward. Nucleic Acids Res.

[30] Ashburner M, Ball CA, Blake JA, Botstein D, Butler H, Cherry JM, Davis AP, Dolinski K, Dwight SS, Eppig JT, Harris MA, Hill DP, Issel-Tarver L (2000). Gene ontology: tool for the unification of biology. The Gene Ontology Consortium. Nat Genet.

[31] Alexa A, Rahnenfuhrer J (2016). topGO: Enrichment Analysis for Gene Ontology. R package version 2240.

[32] Ihaka R, Gentleman R (1996). R: a language for data analysis and graphics. J Comput Graph Stat.

[33] Gentleman R, Carey V, Dudoit S, Ellis B, Gautier L, Gentry J, Huber W, Irizarry R, Rossini A, Smyth G, Zhang J (2003). The Bioconductor Project.

[34] Cancer Genome Atlas Research Network (2013). Comprehensive molecular characterization of clear cell renal cell carcinoma. Nature.

[35] Dos Santos SN, Sheldon H, Pereira JX, Paluch C, Bridges EM, El-Cheikh MC, Harris AL, Bernardes ES (2017). Galectin-3 acts as an angiogenic switch to induce tumor angiogenesis via Jagged-1/Notch activation. Oncotarget.

[36] Sers C, Riethmuller G, Johnson JP (1994). MUC18, a melanoma-progression associated molecule, and its potential role in tumor vascularization and hematogenous spread. Cancer Res.

[37] Lara PC, Lloret M, Clavo B, Apolinario RM, Henriquez-Hernandez LA, Bordon E, Fontes F, Rey A (2009). Severe hypoxia induces chemo-resistance in clinical cervical tumors through MVP over-expression. Radiat Oncol.

[38] Iwashita K, Ikeda R, Takeda Y, Sumizawa T, Furukawa T, Yamaguchi T, Akiyama S, Yamada K (2010). Major vault protein forms complexes with hypoxia-inducible factor (HIF)-1alpha and reduces HIF-1alpha level in ACHN human renal adenocarcinoma cells. Cancer Sci.

[39] Morra L, Rechsteiner M, Casagrande S, Duc Luu V, Santimaria R, Diener PA, Sulser T, Kristiansen G, Schraml P, Moch H, Soltermann A (2011). Relevance of periostin splice variants in renal cell carcinoma. Am J Pathol.

[40] Boysen G, Bausch-Fluck D, Thoma CR, Nowicka AM, Stiehl DP, Cima I, Luu VD, von Teichman A, Hermanns T, Sulser T, Ingold-Heppner B, Fankhauser N, Wenger RH (2012). Identification and functional characterization of pVHL-dependent cell surface proteins in renal cell carcinoma. Neoplasia.

[41] Waalkes S, Atschekzei F, Kramer MW, Hennenlotter J, Vetter G, Becker JU, Stenzl A, Merseburger AS, Schrader AJ, Kuczyk MA, Serth J (2010). Fibronectin 1 mRNA expression correlates with advanced disease in renal cancer. BMC Cancer.

[42] White NM, Masui O, Desouza LV, Krakovska O, Metias S, Romaschin AD, Honey RJ, Stewart R, Pace K, Lee J, Jewett MA, Bjarnason GA, Siu KW (2014). Quantitative proteomic analysis reveals potential diagnostic markers and pathways involved in pathogenesis of renal cell carcinoma. Oncotarget.

[43] Seliger B, Dressler SP, Wang E, Kellner R, Recktenwald CV, Lottspeich F, Marincola FM, Baumgartner M, Atkins D, Lichtenfels R (2009). Combined analysis of transcriptome and proteome data as a tool for the identification of candidate biomarkers in renal cell carcinoma. Proteomics.

[44] Ohno Y, Izumi M, Kawamura T, Nishimura T, Mukai K, Tachibana M (2009). Annexin II represents metastatic potential in clear-cell renal cell carcinoma. Br J Cancer.

[45] Kirschke H, Clausen T, Gohring B, Gunther D, Heucke E, Laube F, Lowe E, Neef H, Papesch H, Peinze S, Plehn G, Rebmann U, Rinne A (1997). Concentrations of lysosomal cysteine proteases are decreased in renal cell carcinoma compared with normal kidney. J Cancer Res Clin Oncol.

[46] Villanueva J, Shaffer DR, Philip J, Chaparro CA, Erdjument-Bromage H, Olshen AB, Fleisher M, Lilja H, Brogi E, Boyd J, Sanchez-Carbayo M, Holland EC, Cordon-Cardo C (2006). Differential exoprotease activities confer tumor-specific serum peptidome patterns. J Clin Invest.

[47] Li X, Lou Z, Li X, Zhou W, Ma M, Cao Y, Geng Y, Bartlam M, Zhang XC, Rao Z (2008). Structure of human cytosolic X-prolyl aminopeptidase: a double Mn(II)-dependent dimeric enzyme with a novel three-domain subunit. J Biol Chem.

[48] Schechter I, Berger A (1968). On the active site of proteases. 3. Mapping the active site of papain; specific peptide inhibitors of papain. Biochem Biophys Res Commun.

[49] Vanhoof G, de Meester I, Hendriks D, Goossens F, van Sande M, Scharpe S, Yaron A (1992). Proline-specific aminopeptidases: potential role in bradykinin degradation. Agents Actions Suppl.

[50] Sprinkle TJ, Caldwell C, Ryan JW (2000). Cloning, chromosomal sublocalization of the human soluble aminopeptidase P gene (XPNPEP1) to 10q25.3 and conservation of the putative proton shuttle and metal ligand binding sites with XPNPEP2. Arch Biochem Biophys.

[51] Taskesen E, Staal FJ, Reinders MJ (2015). An integrated approach of gene expression and DNA-methylation profiles of WNT signaling genes uncovers novel prognostic markers in acute myeloid leukemia. BMC Bioinformatics.

[52] Vanhoof G, De Meester I, van Sande M, Scharpe S, Yaron A (1992). Distribution of proline-specific aminopeptidases in human tissues and body fluids. Eur J Clin Chem Clin Biochem.

[53] Crespigio J, Berbel LCL, Dias MA, Berbel RF, Pereira SS, Pignatelli D, Mazzuco TL (2017 June 6). Von Hippel-Lindau disease: a single gene, several hereditary tumors. J Endocrinol Invest.

[54] Lonser RR, Glenn GM, Walther M, Chew EY, Libutti SK, Linehan WM, Oldfield EH (2003). von Hippel-Lindau disease. Lancet.

[55] Stojanovic M, Goldner B, Ivkovic D (2009). Renal cell carcinoma and arterial hypertension. Clin Exp Nephrol.

[56] Erdos EG, Skidgel RA (1989). Neutral endopeptidase 24.11 (enkephalinase) and related regulators of peptide hormones. FASEB J.

[57] Harbeck HT, Mentlein R (1991). Aminopeptidase P from rat brain. Purification and action on bioactive peptides. Eur J Biochem.

[58] Williams RD, Elliott AY, Stein N, Fraley EE (1978). *In vitro* cultivation of human renal cell cancer. II. Characterization of cell lines. *In Vitro*.

[59] Sjolund J, Johansson M, Manna S, Norin C, Pietras A, Beckman S, Nilsson E, Ljungberg B, Axelson H (2008). Suppression of renal cell carcinoma growth by inhibition of Notch signaling *in vitro* and *in vivo*. J Clin Invest.

[60] Malec V, Coulson JM, Urbe S, Clague MJ (2015). Combined Analyses of the VHL and Hypoxia Signaling Axes in an Isogenic Pairing of Renal Clear Cell Carcinoma Cells. J Proteome Res.

[61] Overall CM, Kleifeld O (2006). Tumour microenvironment - opinion: validating matrix metalloproteinases as drug targets and anti-targets for cancer therapy. Nat Rev Cancer.

[62] Boersema PJ, Aye TT, van Veen TA, Heck AJ, Mohammed S (2008). Triplex protein quantification based on stable isotope labeling by peptide dimethylation applied to cell and tissue lysates. Proteomics.

[63] Rappsilber J, Mann M, Ishihama Y (2007). Protocol for micro-purification, enrichment, pre-fractionation and storage of peptides for proteomics using StageTips. Nat Protoc.

[64] Cox J, Mann M (2008). MaxQuant enables high peptide identification rates, individualized p.p.b.-range mass accuracies and proteome-wide protein quantification. Nat Biotechnol.

[65] UniProt C (2015). UniProt: a hub for protein information. Nucleic Acids Res.

[66] Gomez-Auli A, Hillebrand LE, Biniossek ML, Peters C, Reinheckel T, Schilling O (2016). Impact of cathepsin B on the interstitial fluid proteome of murine breast cancers. Biochimie.

[67] Ritchie ME, Phipson B, Wu D, Hu Y, Law CW, Shi W, Smyth GK (2015). limma powers differential expression analyses for RNA-sequencing and microarray studies. Nucleic Acids Res.

[68] Sigloch FC, Knopf JD, Weisser J, Gomez-Auli A, Biniossek ML, Petrera A, Schilling O (2016). Proteomic analysis of silenced cathepsin B expression suggests non-proteolytic cathepsin B functionality. Biochim Biophys Acta.

[69] Tholen S, Biniossek ML, Gessler AL, Muller S, Weisser J, Kizhakkedathu JN, Reinheckel T, Schilling O (2011). Contribution of cathepsin L to secretome composition and cleavage pattern of mouse embryonic fibroblasts. Biol Chem.

[70] Sigloch FC, Burk UC, Biniossek ML, Brabletz T, Schilling O (2015). miR-200c dampens cancer cell migration via regulation of protein kinase A subunits. Oncotarget.

[71] Vizcaino JA, Csordas A, del-Toro N, Dianes JA, Griss J, Lavidas I, Mayer G, Perez-Riverol Y, Reisinger F, Ternent T, Xu QW, Wang R, Hermjakob H (2016). 2016 update of the PRIDE database and its related tools. Nucleic Acids Res.

