# Supplementary material for: Proteome profiling of clear cell renal cell carcinoma in von Hippel-Lindau patients highlights upregulation of Xaa-Pro aminopeptidase-1, an anti-proliferative and anti-migratory exoprotease
Source: Oncotarget. 2017 Oct 19;8(59):100066–78. doi: 10.18632/oncotarget.21929 (PMC5725003; doi:10.18632/oncotarget.21929)
Supplement: Supplementary file 1 [file oncotarget-08-100066-s001.pdf]

Proteome profiling of clear cell renal cell carcinoma in von Hippel-Lindau patients highlights upregulation of Xaa-Pro aminopeptidase-1, an anti-proliferative and anti-migratory exoprotease

SUPPLEMENTARY MATERIALS

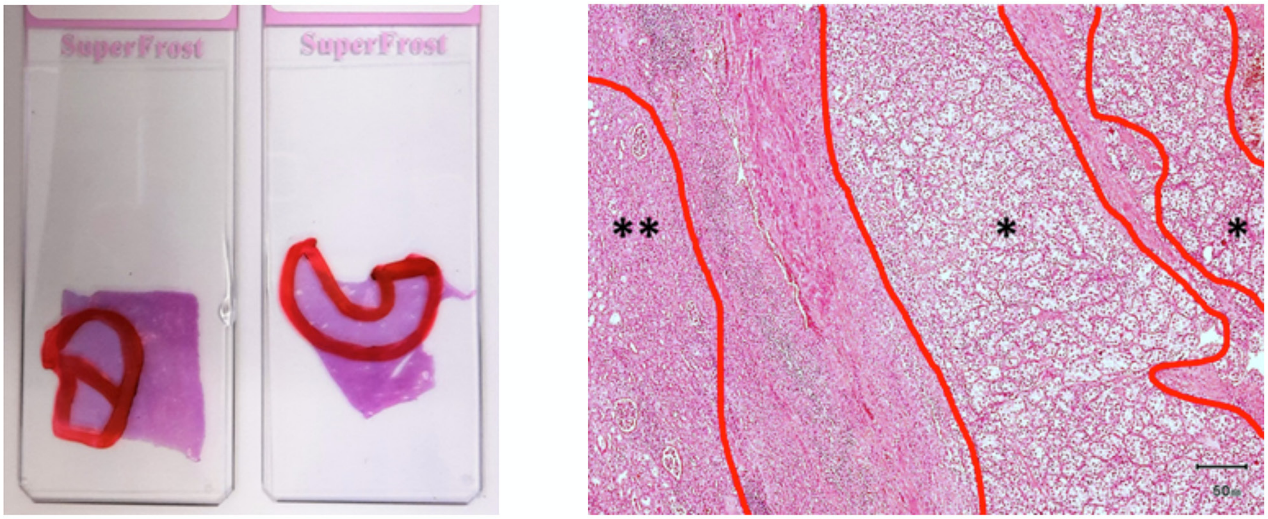

* ccRCC area

** non-neo-plastic kidney

Supplementary Figure 1: Exemplary photomicrograph of a ccRCC with areas of hemorrhage, fibrosis, and lymphocytic infiltration, which also extend into adjacent non-neoplastic kidney. This areas were marked on the HE stained FFPE section under the microscope and removed by macrodissection

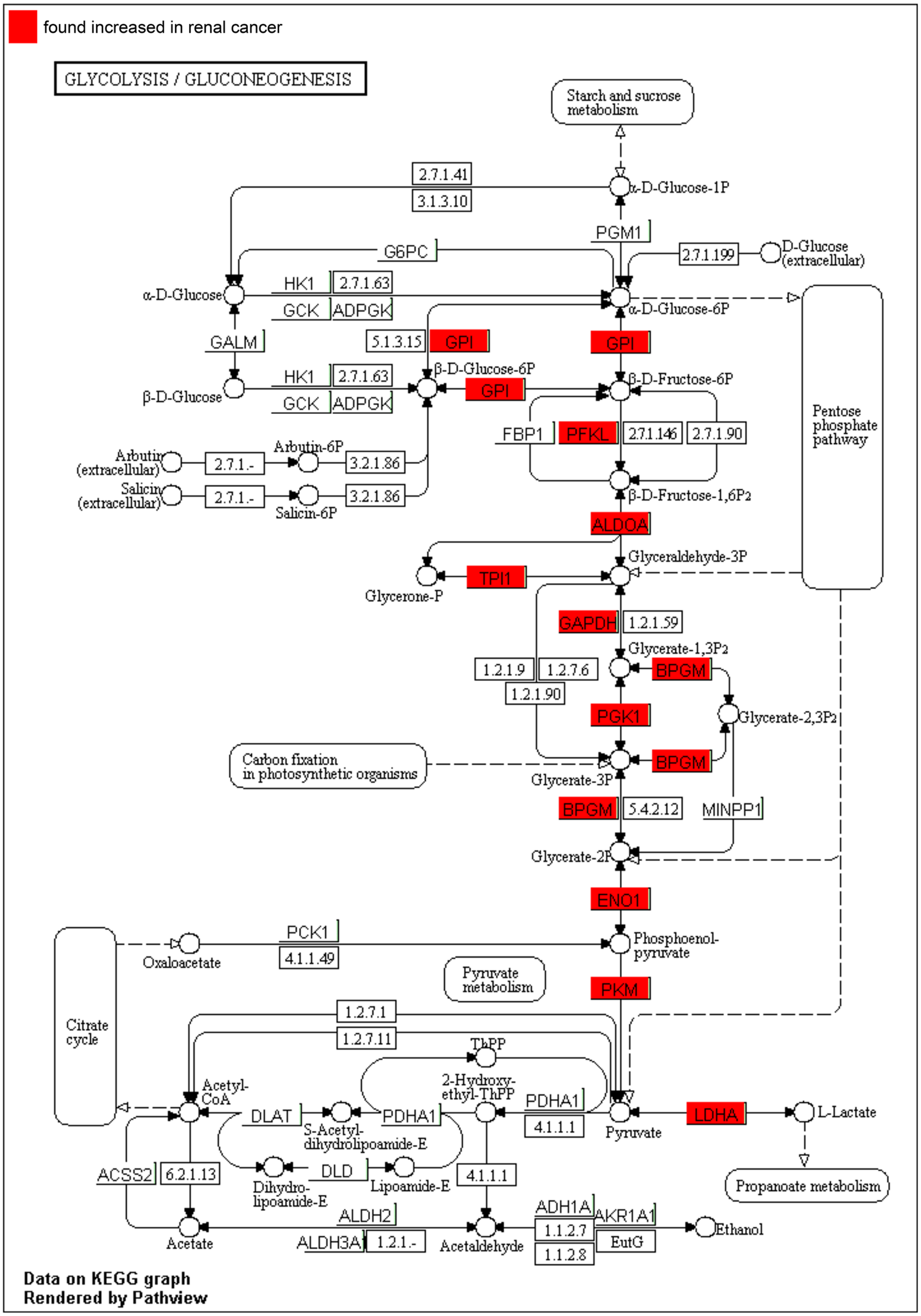

Supplementary Figure 2: Mapping of significantly down-regulated glycolytic enzymes (red) on the glycolysis pathway as depicted by the Kyoto Encyclopedia of Genes and Genomes (KEGG).

Supplementary Table 1: Proteins that were identified and quantified in at least six of the eight cases

See Supplementary File 1
